# A TME-activated photothermal agent with photodegradability for accurate breast tumor photothermal therapy†

**DOI:** 10.1039/d5ra03064a

**Published:** 2025-07-15

**Authors:** Weiqian Zhang, Weiqing Yue, Xiaoyi Shi, Liyun Lv, Zhijie Fang, Jie Li

**Affiliations:** a State Key Laboratory of Flexible Electronics (LoFE), Institute of Advanced Materials (IAM), School of Flexible Electronics (Future Technologies), Nanjing Tech University (NanjingTech) Nanjing 211816 China iamjli@njtech.edu.cn iamxmlu@njtech.edu.cn

## Abstract

Photothermal therapy (PTT) has garnered significant attention due to its high controllability and minimal invasiveness. However, PTT remains hindered by the non-specificity and biosafety concerns associated with traditional photothermal agents. In this work, we successfully developed a tumor microenvironment (TME)-activated intelligent photothermal agent, IR780-PDA, which exhibits a GSH-activated photophysical transformation. The absorption peak of IR780-PDA red-shifts from 640 nm to 790 nm, markedly enhancing its photothermal conversion efficiency under near-infrared laser irradiation. Additionally, IR780-PDA possesses dual-wavelength photoacoustic imaging capabilities at 640 nm and 790 nm, facilitating accurate tumor localization and real-time imaging monitoring. Under conditions of high GSH levels and 808 nm laser irradiation, IR780-PDA undergoes photodegradation, promoting efficient *in vivo* metabolism and improving biosafety. Animal experiments demonstrated that IR780-PDA achieves specific tumor enrichment in breast cancer models and completely eradicates tumors, showcasing excellent therapeutic efficacy and good biocompatibility.

## Abstract 2

Through the integration of a GSH-activated design, IR780-PDA exhibits a pronounced redshift in absorption within the tumor microenvironment, enabling enhanced photothermal precision under NIR irradiation for photothermal therapy.

## Graphical abstract

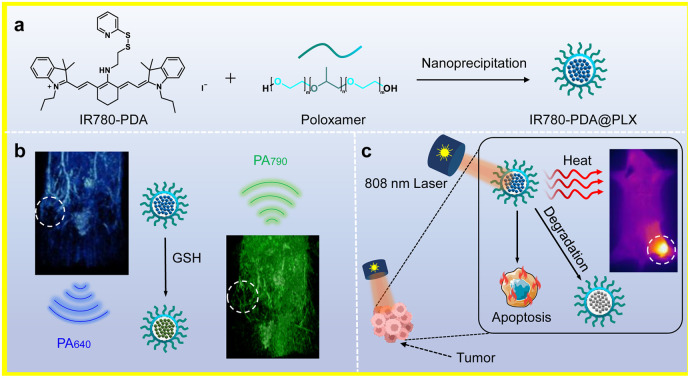

Photothermal therapy (PTT), as an emerging therapeutic modality, relies on photothermal agents (PTAs) to convert light energy into localized heat,^1^ inducing irreversible damage to tumor cells.^2–5^ Compared with conventional radiotherapy and chemotherapy, PTT offers several notable advantages, including minimal side effects and high spatial resolution.^6–9^ However, the practical implementation of PTT continues to be constrained by several critical challenges. Traditional PTAs often suffer from limited biocompatibility, suboptimal photothermal conversion efficiency (PCE), and insufficient tumor accumulation.^10^ Moreover, most PTAs lack adequate tumor-targeting specificity and tend to exhibit nonspecific distribution and activation in normal tissues, leading to off-target thermal damage.^11^ This uncontrolled heating effect not only increases treatment-associated side effects but also poses a substantial barrier to the safe and precise application of PTT.^12^

The unique characteristics of the tumor microenvironment (TME)^13–15^ have provided new opportunities for enhancing the specificity and biosafety of PTT.^16,17^ TME composed of tumor cells and their surrounding immune cells, stromal cells, vasculature, and extracellular matrix, is characterized by distinct physicochemical features such as persistent hypoxia,^18–20^ acidic pH,^21–23^ elevated glutathione (GSH) levels,^24^ and reactive oxygen species (ROS) imbalance.^25^ Based on these pathological characteristics, activatable intelligent PTAs with TME-responsive capabilities can be designed to achieve selective activation at tumor sites.^26–28^ This strategy enables for spatial control of heat generation and precise therapeutic release, minimizing collateral thermal damage to normal tissues.^29^ In addition to tumor-selective activation, the degradability of PTAs is another key factor in improving the biosafety profile of PTT. The incorporation of controllable degradable structural units or exogenous stimulus-responsive mechanisms, PTAs can be designed to undergo spontaneous or induced degradation post-treatment.^30,31^ This approach effectively minimizes their long-term retention in the body and potential accumulation toxicity, enhancing the biosafety and controllability of PTT.

Herein, we developed an intelligent photothermal agent, IR780-PDA, capable of TME responding. In the TME with elevated GSH levels, IR780-PDA exhibits a significant redshift in absorption, with its main absorption peak shifting from 640 nm to 790 nm. This spectral shift markedly improves its PCE under 808 nm laser irradiation, enabling precise and efficient PTT while minimizing non-specific thermal damage to normal tissues during treatment. In addition, IR780-PDA exhibits dual-wavelength radiometric photoacoustic (PA) imaging capabilities, enabling simultaneous generation of PA signals at 640 nm and 790 nm, which significantly enhances imaging spatial resolution and tumor specificity, aiding in the precise localization of tumor region. IR780-PDA exhibited instability in GSH-rich environments and under 808 nm laser irradiation, resulting in time-dependent degradation during treatment and significantly improving biosafety. In breast cancer mouse model demonstrated that IR780-PDA could effectively accumulate in tumor tissues and markedly suppress tumor growth upon 808 nm laser irradiation, showcasing superior therapeutic efficacy and excellent biocompatibility (Scheme 1).

**Scheme 1:**
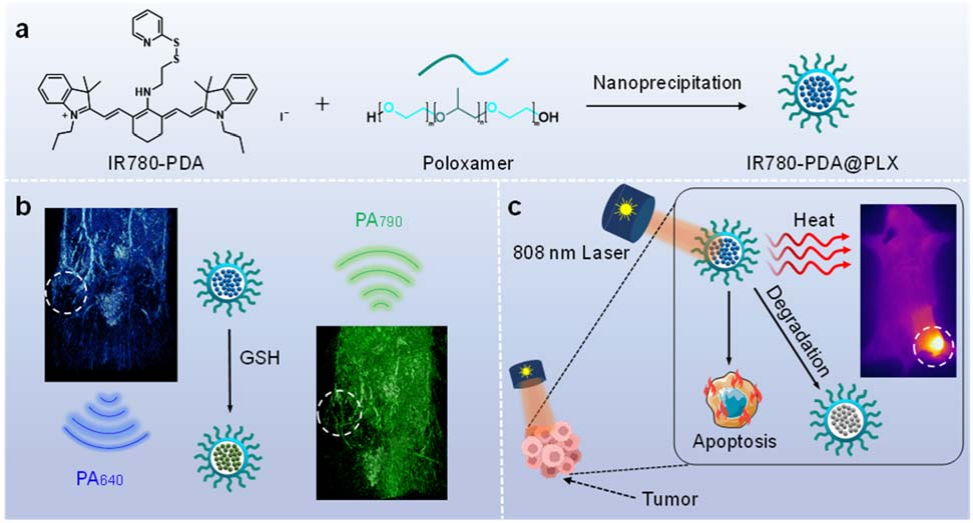
(a) Synthesis and chemical structure of IR780-PDA. (b) Schematic illustration of the nanoparticle (IR780-PDA@PLX) preparation process. (c) PA imaging and PTT for breast cancer in mice models.

The TME-activatable IR780-PDA was successfully synthesized as a brownish-yellow solid powder (Fig. S1†). Its chemical structure was systematically characterized using proton nuclear magnetic resonance (^1^H NMR), carbon nuclear magnetic resonance (^13^C NMR), and high-resolution mass spectrometry (HR-MS) (Fig. S2–S4†). IR780-PDA is derived from the near-infrared absorbing dye IR780 as the parent structure, where its active chlorine sites were modified with amino groups to introduce a thiol-responsive disulfide bond functional group (–S–S–PDA). This molecular design enables controllable structural transformation in the presence of glutathione (GSH): the disulfide bond can be specifically reduced and cleaved by GSH, generating an intermediate containing free thiol groups (–SH), which subsequently undergoes nucleophilic substitution with secondary amine sites to form the restructured product IR780-S-NH_2_.

To improve its biological stability and dispersibility, IR780-PDA was encapsulated with poloxamer to form nanoparticles designated as IR780-PDA@PLX. Transmission electron microscopy (TEM) and dynamic light scattering (DLS) analyses revealed that these nanoparticles were spherical in shape, with an average diameter of approximately 80 nm (Fig. 1a). To investigate the responsiveness of IR780-PDA@PLX to GSH, optical absorption experiments were performed in PBS at pH 7.4. The results demonstrated that untreated IR780-PDA@PLX exhibited absorption peak at 640 nm, and the solution appeared blue. As the GSH concentration increased incrementally from 0 to 1000 μM, the absorption peak at 640 nm progressively diminished, while the characteristic absorption of IR780-S-NH_2_ at 790 nm concurrently increased. This transition resulted in a visible color change of the solution from blue to green (Fig. 1b and c). Through calculating the ratio of absorption intensities at 790 nm and 640 nm (Ab_790_/Ab_640_) under various GSH concentrations, it was observed that this ratio reached a stable plateau when the GSH concentration increased to 60 mM, indicating that the system exhibits a saturation response characteristic to GSH concentration (Fig. S5†). Further investigation into the dynamic time-dependent behavior of the Ab_790_/Ab_640_ ratio induced by GSH was conducted (Fig. 1d). The results demonstrated that the Ab_790_/Ab_640_ ratio stabilized approximately 1 h after the reaction commenced, suggesting that the reaction process exhibited excellent temporal controllability. To verify the specificity of IR780-PDA@PLX, reaction assessments were performed using common amino acids as controls. The experimental results revealed that IR780-PDA@PLX exhibited no significant response to the interfering substances, whereas a marked increase in the Ab_790_/Ab_640_ ratio was observed in the presence of GSH (Fig. S6†). Furthermore, the reaction behavior of GSH with IR780-PDA@PLX under varying pH conditions was systematically examined (Fig. S7†), and the findings indicated that the reaction process was largely unaffected by pH changes, demonstrating excellent pH stability.

**Fig. 1:**
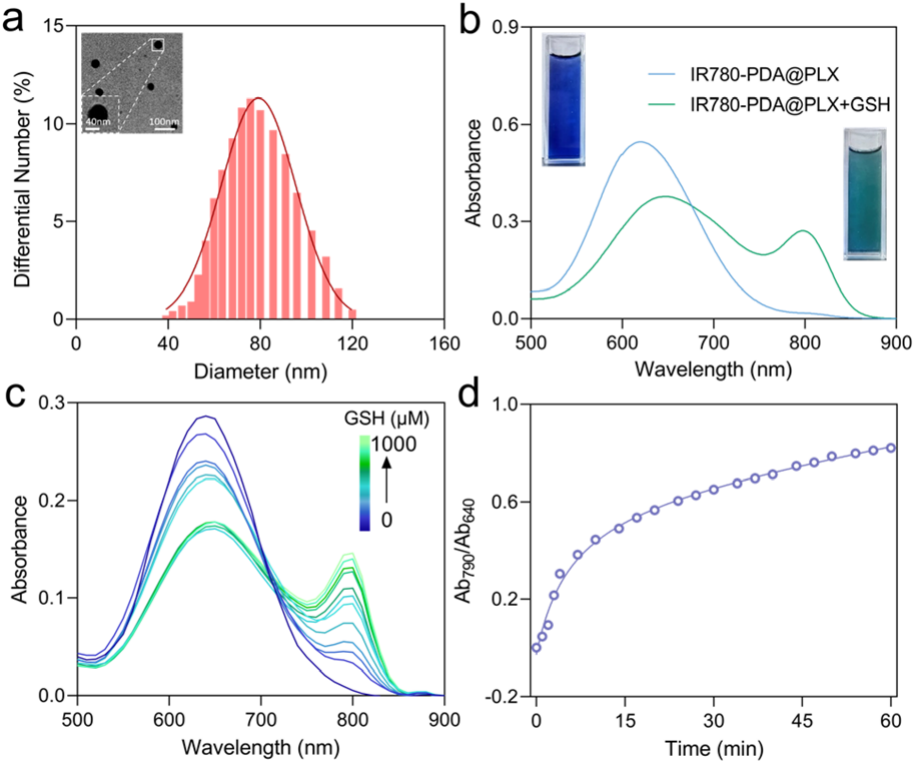
(a) The DLS and TEM (insert Fig.) image of IR780-PDA@PLX. Scale bar: 100 nm. (b) The absorption spectra and solution color changes of IR780-PDA@PLX before and after reacting with GSH. (c) Absorption spectra of IR780-PDA@PLX upon addition of GSH (0–1000 μM) in PBS. (d) The ratiometric absorbance intensities kinetics curves of reaction between IR780-PDA@PLX and GSH (1000 μM).

To verify the PA imaging capability of IR780-PDA@PLX, the PA imaging of GSH-activated IR780-PDA@PLX was first demonstrated in solution. Subsequently, PA imaging at two wavelengths (640 nm and 790 nm) was performed after incubating IR780-PDA@PLX with GSH, with the signals displayed in blue (PA_640_) and green (PA_790_), respectively. Upon adding GSH to IR780-PDA@PLX, the blue signal (PA_640_) was significantly attenuated compared to the control group, whereas the green signal (PA_790_) exhibited a marked increase (Fig. 2a). A strong linear correlation was observed between the PA intensity ratio (PA_790_/PA_640_) and the GSH concentration (Fig. 2b). Time-dependent PA imaging revealed that as the incubation time with GSH increased, the blue signal (PA_640_) gradually decreased, while the green signal (PA_790_) significantly increased (Fig. 2c). The results of the PA intensity ratio (PA_790_/PA_640_) indicated that the increase in PA790/PA640 stabilized approximately 1 h after GSH addition to IR780-PDA@PLX. This observation was consistent with the kinetic behaviour observed in the spectral changes of GSH-activated IR780-PDA@PLX (Fig. 2d).

**Fig. 2:**
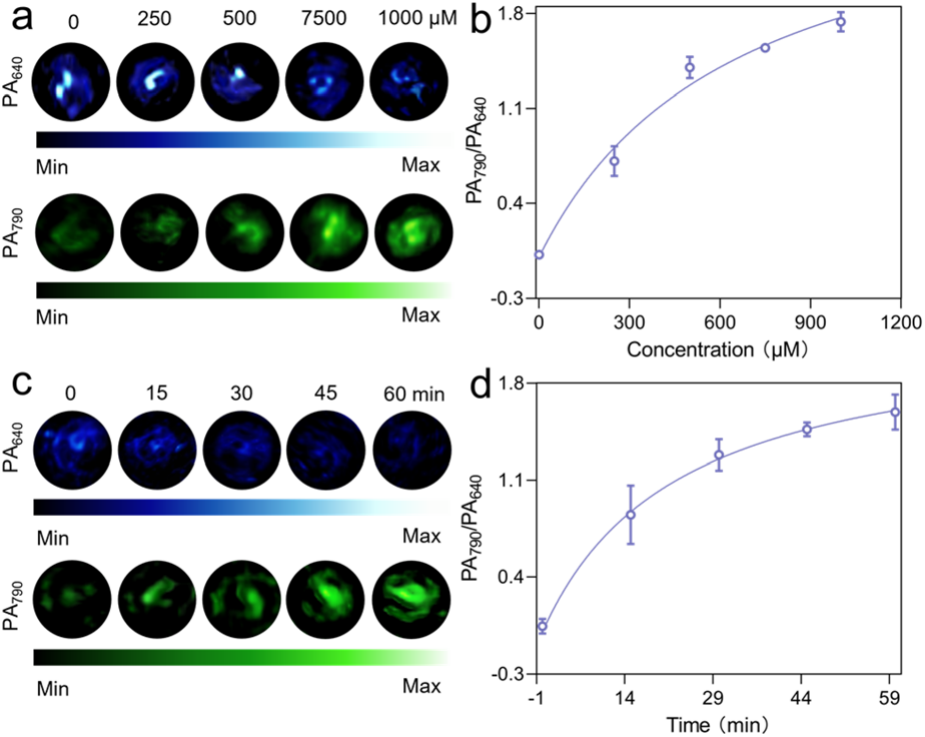
(a) PA images (640 nm and 790 nm) of IR780-PDA@PLX reacting with different concentrations of GSH (0, 250, 500, 750 and 1000 μM). (b) Ratiometric PA intensities (PA_790_/PA_640_) of different GSH concentrations reacting with IR780-PDA@PLX. (c) Images of PA signal changes at 640 nm and 790 nm at the time of cleavage. (d) Ratiometric PA intensities (PA_790_/PA_640_) of GSH reacting with IR780-PDA@PLX at different times.

Subsequently, given the near-infrared absorption wavelength of IR780-PDA@PLX at 790 nm after GSH activation, an 808 nm laser was employed as the excitation light source to assess the photothermal performance of GSH-activated IR780-PDA@PLX. Through theoretical calculations, the PCE of GSH-activated IR780-PDA@PLX under 808 nm laser irradiation was determined to be 43.65% (Fig. 3a). To maintain the temperature required for effective PTT during a 10-minute treatment, the laser power was limited to 0.5 W cm^−2^ (the maximum permissible exposure), and the IR780-PDA@PLX concentration was adjusted accordingly. The experimental results demonstrated that GSH-activated IR780-PDA@PLX could sustain a temperature above 42 °C for 816 s (Fig. 3b). The results of photostability showed that the maximum temperature of GSH activated IR780-PDA@PLX could reach was close to 76 °C during the first laser irradiation. And the maximum temperature could only reach 67 °C during the second irradiation, and continued to drop to 58 °C during the third irradiation. This result show that GSH activated IR780-PDA@PLX has poor photostability and certain photodegradation properties (Fig. S8†). Cell viability following treatment with IR780-PDA@PLX was assessed using the Cell Counting Kit-8 (CCK-8). IR780-PDA@PLX exhibited minimal cytotoxicity, maintaining a cell survival rate above 80% even at a high concentration of 4 mg mL^−1^. Upon exposure to an 808 nm laser, cell viability decreased significantly, with the half-maximal inhibitory concentration (IC50) of IR780-PDA@PLX determined to be 0.15 mg mL^−1^ (Fig. 3c). Apoptosis analysis (using calcein AM for live cells and propidium iodide (PI) for apoptotic cells) further confirmed the cytotoxicity of IR780-PDA@PLX upon 808 nm laser exposure. In the non-irradiated group (Group 1), no red fluorescence from PI-stained dead cells was detected. However, after irradiation with an 808 nm laser (Group 2), a significant enhancement in red fluorescence was observed (Fig. 3d).

**Fig. 3:**
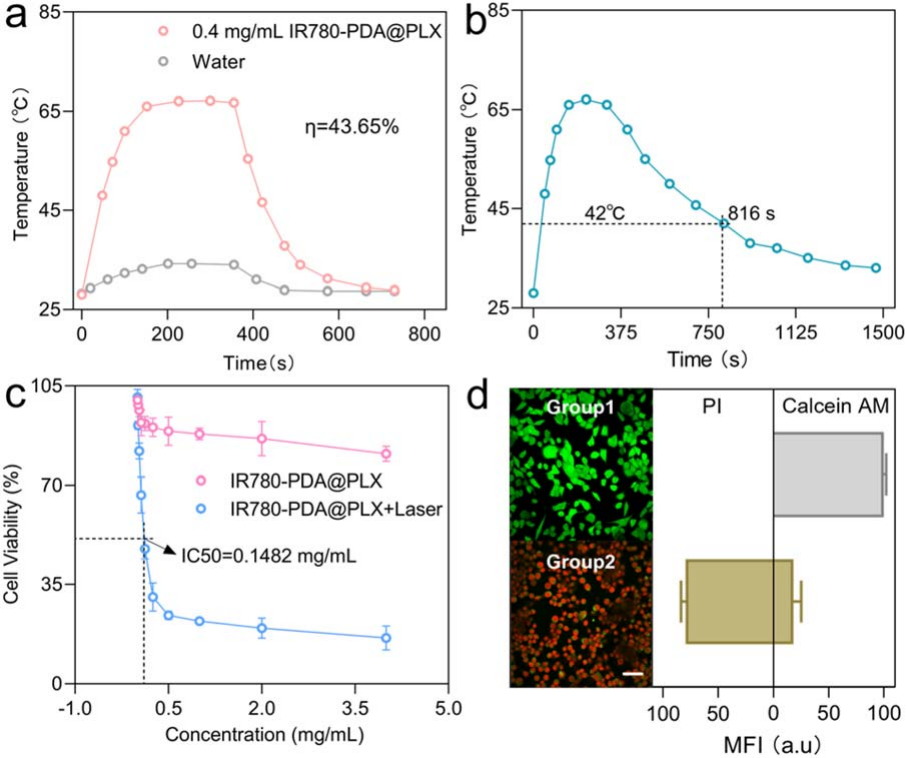
(a) The photothermal conversion efficiencies under laser irradiation at 808 nm. (b) Photothermic heating curves of IR780-PDA@PLX in presence of GSH under laser irradiation at 808 nm. (c) Cytotoxicity of IR780-PDA@PLX with and without 808 nm irradiation for 10 min. (d) Cells apoptosis evaluation of 4T1 cell with calcein AM/PI assay staining. Green channel: calcein AM; red: PI. Scale bar = 100 μm.

The *in situ* breast cancer mouse model was employed to systematically evaluate the PA imaging capability of IR780-PDA@PLX in the tumor region. PA images were acquired at two excitation wavelengths (640 nm and 790 nm), with the signals displayed in blue and green pseudo-colors, respectively (Fig. 4a and S9†). The experimental results demonstrated that the ΔPA_640_ signal intensity in the tumor region gradually increased after injection and reached its peak at 12 h post-injection; in contrast, the ΔPA_790_ signal intensity peaked approximately 8 h post-injection and subsequently remained stable (Fig. S10†). Further quantitative analysis of the PA signal ratio (ΔPA_790_/ΔPA_640_) revealed that this ratio progressively increased over time and reached its maximum at 12 h post-injection (Fig. 4b). These findings suggest that IR780-PDA@PLX enables highly timely and specific radiometric PA imaging. *In vivo* imaging was further conducted to monitor the long-term accumulation and metabolism of IR780-PDA@PLX in mice. The results showed that the fluorescence intensity at the tumor site gradually increased over time, reaching a maximum at 12 h and remaining stable up to 96 h. In contrast, the fluorescence intensity in the liver peaked at 8 h and then exhibited a continuous decline over the 96-h period. Finally, ex vivo imaging of dissected organs and tumors confirmed that the metabolic clearance of IR780-PDA@PLX was primarily mediated through the liver and spleen (Fig. S11†).

**Fig. 4:**
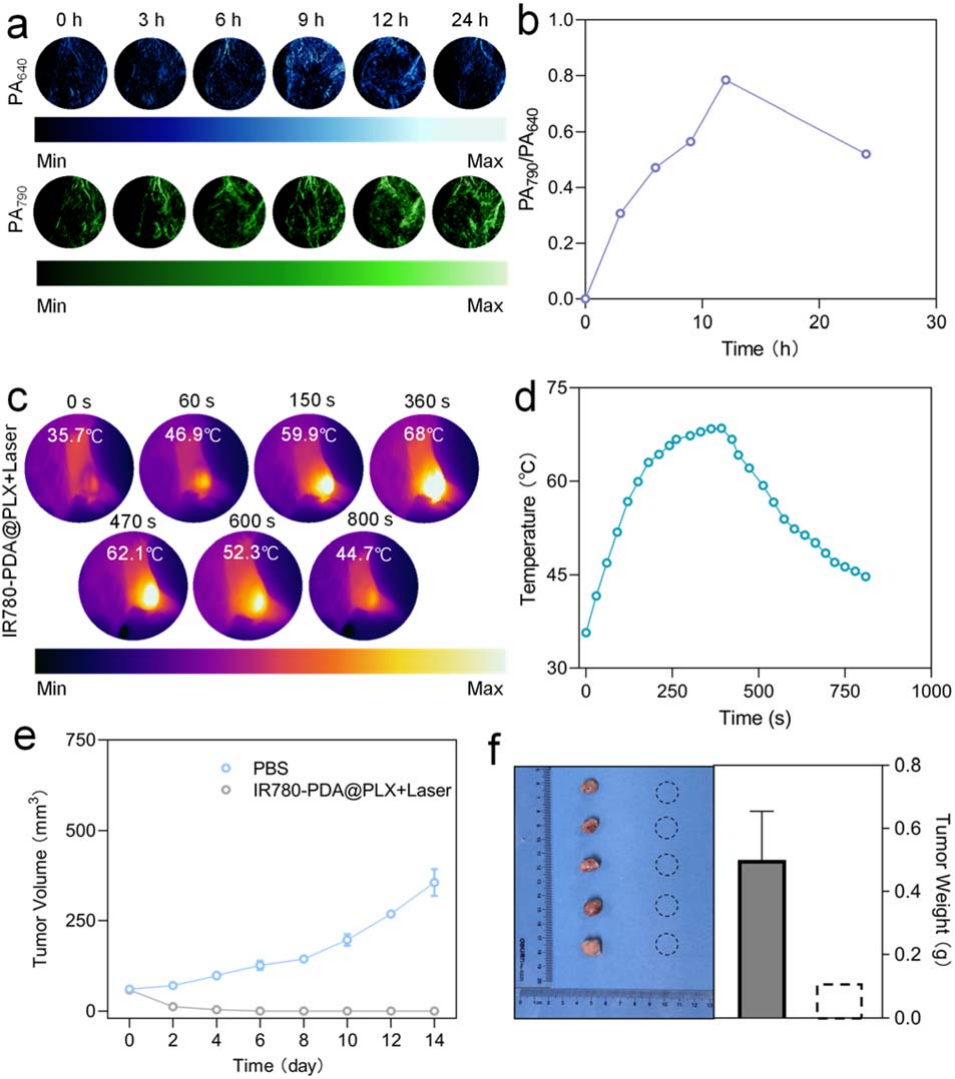
(a) PA images of 4T1 tumor in nude mouse before and after systemic administration for 0, 3, 6, 9, 12 and 24 h. PA signal was collected under a pulsed laser turning to 640 or 790 nm. (b) Ratiometric PA intensities (PA_790_/PA_640_) as a function of postinjection time. (c) Thermal imaging of tumor site of mice following IR780-PDA@PLX injection under laser irradiation (0.5 W cm^−2^) within 800 s. (d) Time-dependent temperature rise curve at the tumor site in mice. (e) 4T1 tumor volume variation curves in treatment groups. *n* = 5 biologically independent animals. (f) Photographs of removed tumors and tumor weights after the therapeutics. *n* = 5 biologically independent animals.

To assess the thermal response performance of IR780-PDA@PLX in PTT, 808 nm laser was applied to tumor region 12 h post-tail vein injection. Infrared thermal imaging monitoring revealed that the temperature at the tumor site rapidly increased to approximately 42 °C within 30 s of irradiation, demonstrating successful activation of IR780-PDA@PLX in TME and efficient conversion of light energy into heat energy (Fig. 4c and S12†). Given that IR780-PDA@PLX exhibits GSH-triggered structural dissociation and photodegradation properties, its thermal effect may diminish over extended irradiation periods. Therefore, it is essential to further evaluate its thermal stability and therapeutic efficacy under continuous laser irradiation (Fig. 4d). The results indicated that the local tumor temperature remained stable at 44.7 °C after continuous irradiation for 800 s, significantly exceeding the threshold temperature of 42 °C required for effective tumor ablation. This finding confirms that IR780-PDA@PLX satisfies the requirements for temperature persistence and therapeutic intensity necessary for effective PTT.

The *in vivo* therapeutic efficacy of IR780-PDA@PLX was systematically evaluated. Mice were divided into groups and subjected to different treatments, followed by continuous monitoring of tumor growth dynamics (Fig. 4e). The results demonstrated that tumors in the control group (treated with PBS) exhibited rapid growth, with the final tumor volume increasing to approximately five times the initial volume by the end of the treatment period. In contrast, complete tumor regression was observed in mice treated with IR780-PDA@PLX injection combined with 808 nm laser irradiation, with no visible residual lesions detected post-treatment (Fig. 4f). Upon euthanasia, tumor tissues were collected and weighed. Notably, no tumor tissue was detected in the treatment group, further validating the potent anti-tumor efficacy of IR780-PDA@PLX. Additionally, histopathological analysis of major organs (heart, liver, spleen, lung, and kidney) revealed no significant signs of tissue damage, inflammatory infiltration, or necrosis (Fig. S13†). Furthermore, body weight measurements indicated stable weight maintenance across all experimental groups throughout the treatment period, without significant fluctuations, underscoring the excellent biocompatibility and low toxicity of IR780-PDA@PLX (Fig. S14†). During the treatment process, blood biochemical indicators were closely monitored, including alanine aminotransferase (ALT), aspartate aminotransferase (AST), lactate dehydrogenase (LDH), creatine kinase (CK), blood urea nitrogen (BUN), and creatinine (CRE). The results demonstrated that no statistically significant differences were observed in these parameters between the treatment group and the PBS control group. Further substantiates the excellent biocompatibility of IR780-PDA@PLX (Fig. S15†).

In this work, we successfully developed an intelligent photothermal agent, IR780-PDA@PLX, with TME-activated characteristics. Upon stimulation by GSH, IR780-PDA@PLX undergoes significant changes in its optical properties, with the main absorption peak red-shifting from 640 nm to 790 nm, enhancing the PCE of IR780-PDA@PLX under near-infrared laser irradiation (808 nm), effectively minimizing non-specific thermal damage to surrounding normal tissues. IR780-PDA@PLX exhibits dual-band ratio-type PA imaging capabilities, generating PA signals at 640 nm and 790 nm, respectively. This feature improves the spatial resolution of imaging and enhances the accuracy of tumor-targeted localization, providing robust support for preoperative diagnosis and intraoperative navigation. In a breast cancer mouse model, IR780-PDA@PLX demonstrates effective tumor accumulation after intravenous injection and achieves significant tumor suppression under 808 nm laser irradiation. In addition, IR780-PDA@PLX possesses favorable photodegradability, enabling its degradation under light exposure after treatment, which reduces long-term *in vivo* accumulation and enhances overall biosafety.

To further enhance the imaging and therapeutic capabilities of TME-activated treatments, we provide a brief outlook on the future prospects of such probes. Since IR780-PDA primarily operates within the near-infrared (NIR) windows, extending its responsiveness into the far-infrared (FIR) region could enable deeper tissue penetration and improved imaging quality. This extension may be achieved through molecular engineering strategies to red-shift the absorption spectrum or by integrating IR780-PDA with FIR-responsive inorganic materials. The combination of organic and inorganic components holds promise for the development of next-generation TME-activated agents with longer-wavelength responsiveness, thereby enhancing both imaging precision and therapeutic efficacy.

## Ethics approval

All animal experiments were performed with the permission of the Animal Ethics Committee of Jiangsu Keygen Biotech Co., Ltd (China), according to the guidelines approved by the Jiangsu Administration of Experimental Animals (Approval Number: IACUC-002-24).

## Conflicts of interest

There are no conflicts to declare.

## Supplementary Material

RA-015-D5RA03064A-s001

## Data Availability

The datasets supporting this work including raw experimental data, characterization results, and computational analyses, are available from the corresponding author upon reasonable request. ESI data related to this article, including detailed experimental procedures and additional figures, are provided in the ESI.†
