# Supplementary material for: A TME-activated photothermal agent with photodegradability for accurate breast tumor photothermal therapy
Source: RSC Adv. 2025 Jul 15;15(31):25062–6. doi: 10.1039/d5ra03064a (PMC12262166; doi:10.1039/d5ra03064a)
Supplement: RA-015-D5RA03064A-s001 [file RA-015-D5RA03064A-s001.pdf]

Supplementary Information (SI) for RSC Advances.
This journal is © The Royal Society of Chemistry 2025

A TME-activated photothermal agent with photodegradability for accurate breast tumor photothermal therapy

Experimental Section

1. Materials and Reagents

Triethylamine (TEA), anhydrous tetrahydrofuran (THF), dichloromethane (DCM), petroleum ether (PE), ethyl acetate (EA) were purchased from Nanjing Evening Glass Instrument Co. Hexane (Hex), anhydrous sodium sulfate (Na_2SO_4), potassium hydroxide (KOH) were purchased from Shanghai BiDe Pharmaceutical Co. Glutathione (GSH), Iodide IR780, 2-(2-pyridyl disulfide) ethylamine hydrochloride was purchased from Shanghai Titan Technology Co. Female white rats (BALB/c, 4-6 weeks) purchased from Nanjing Kaiji Biotechnology Co. were used. The guidelines and protocols for use were carried out as approved above.

2. Instrumentations

A transmission electron microscope (JEM2010) was used to obtain transmission electron microscopy (TEM) images. Nuclear magnetic resonance (NMR) spectra were obtained on a nuclear magnetic resonance spectrometer 400-MHz (Nippon Electron Co., Ltd.). Mass spectra were obtained on an Apex Ultra high-resolution mass spectrometer. Absorption spectra were determined using a UV-visible near-infrared spectrophotometer UV-1780 (Perkin Elmer). Photothermal imaging was obtained on an InGaAs NIR camera (Tianying Photonics, China). In vivo and in vitro PA imaging was performed on a photoacoustic imager (NeXuS 128).

3. Synthesis of IR780-PDA

25 mg of 2-(2-pyridyldithio) ethylamine hydrochloride was completely dissolved in dichloromethane. Subsequently, 5 mL of potassium hydroxide solution was added, and the mixture was transferred to a separatory funnel and thoroughly shaken. The upper aqueous layer was discarded. An appropriate amount of anhydrous sodium sulfate was then added to the remaining organic phase to remove residual moisture. The

solution was washed with hexane and concentrated under reduced pressure to yield a yellow oily liquid, referred to as PDA.

Under N_2 atmosphere, 20 mg of PDA was dissolved in anhydrous tetrahydrofuran (THF). Then, 36 mg of IR780 iodide and 200 μL of triethylamine were added to adjust the reaction solution to a weakly basic pH value. The reaction mixture was maintained at 40 °C for 12 h. After completion, the solvent was removed by rotary evaporation. The crude product was subsequently purified by column chromatography to afford a blue solid. (32 mg, 60% yield). ^1H NMR (400 MHz, $CDCl_3$) δ: 8.55-7.26 (m, 7H), 6.87 (s, 8H), 5.65 (s, 1H), 4.20-2.47 (m,13H), 1.80 (s, 6H), 1.57 (s, 2H), 1.53-1.25 (m,10H), 1.04 (s, 6H). ^{13}C NMR (101 MHz, $CDCl_3$) δ: 149.68, 139.00, 137.56, 131.04, 128.93, 128.16, 122.15, 108.63, 77.36, 65.67, 32.02, 30.64, 29.79, 29.30, 29.14, 26.86, 25.17, 21.71, 20.22, 19.27, 14.23, 13.83, 11.86, 1.11, 0.09. HRMS (m/z): 689.3693.

4. Preparation of IR780-PDA@PLX

1 mg of IR780-PDA was completely dissolved in 200 μL of tetrahydrofuran (THF). Separately, 10 mg of poloxamer was fully dissolved in 10 mL of deionized water. Under sonication, the IR780-PDA/THF solution was added dropwise to the aqueous poloxamer solution. The resulting mixture was stirred on a magnetic stirrer for 12 h at room temperature to allow complete evaporation of THF. The final suspension was then centrifuged and concentrated to a final volume of 1 mL.

5. Characterization of IR780-PDA@PLX

5.1 Absorption Spectrum Test of IR780-PDA@PLX

10 μM solution of IR780-PDA@PLX was prepared in an appropriate solvent. The absorption profile of the sample was recorded using a UV-visible-near-infrared (UV-vis-NIR) spectrophotometer, scanning across the relevant wavelength range to characterize the optical properties and identify the characteristic absorption peaks of IR780-PDA@PLX.

5.2 Cleavage behavior of IR780-PDA@PLX under varying GSH concentrations and reaction times.

10 μM IR780-PDA@PLX solution was prepared as the reaction system. For

concentration-dependent studies, 1 mL aliquots of the IR780-PDA@PLX solution were mixed with varying concentrations of GSH at predetermined molar ratios. Each mixture was thoroughly vortexed and incubated at room temperature for 1 h. After incubation, the absorption spectra were recorded using a UV-vis-NIR spectrophotometer to assess changes.

For the time-dependent cleavage analysis, 1 mL of IR780-PDA@PLX solution was treated with GSH at a fixed concentration ratio of 1:100. The reaction mixture was gently agitated and maintained at room temperature. UV-vis-NIR absorbance at 790 nm (A_{790}) and 640 nm (A_{640}) was measured at 10-minute intervals.

5.3 Selectivity evaluation of IR780-PDA@PLX

To assess the selectivity of IR780-PDA@PLX toward GSH over other amino acids, a 10 μM IR780-PDA@PLX solution was prepared as the test sample. For each selectivity assay, 2 mL of the IR780-PDA@PLX solution was mixed with 100 μM of various amino acids (e.g., cysteine, homocysteine, alanine, serine, etc.) and incubated at room temperature for 1 h to allow sufficient interaction. Following incubation, the absorbance of the reaction mixtures was measured at 790 nm and 640 nm using a UV–Vis spectrophotometer. The changes in absorbance (A_{790} and A_{640}) were compared to evaluate the response specificity of IR780-PDA@PLX toward GSH relative to other biologically relevant amino acids.

6. In vitro photothermal imaging

200 μL of activated 0.4 mg/mL IR780-PDA@PLX solution was used as the reaction solution, and the liquid was exposed to an 808 nm laser with a power density of 0.5 mW/cm^2, and when the liquid reached the highest stable temperature, the laser was turned off and allowed to cool down naturally to room temperature. In addition, 200 μL of aqueous solution was taken for the same operation. The temperature rise and temperature profile were recorded and the corresponding conversion efficiency was calculated according to the photothermal conversion efficiency.

To study the photothermal properties of the activated IR780-PDA@PLX aqueous solution, we employed the following method: the activated material solution was placed

under an 808 nm laser, with pure water used as a control, and irradiated for approximately 10 min. The temperature profiles and images were precisely recorded with a thermal imaging camera. To measure the photothermal conversion efficiency of the activated material, the test solution was irradiated with an 808 nm laser at a power density of 0.5 W/cm^2. During irradiation, once the temperature of the test solution reached its maximum and stabilized, the laser was immediately turned off to allow natural cooling to room temperature. This procedure was used to determine the photothermal conversion efficiency of IR780-PDA@PLX, with deionized water serving as the control. The photothermal conversion efficiencies (η) was determined according to the formula:

$$\eta = \frac{hS(T_{max} - T_{Surr}) - Q_{Dis}}{I(1 - 10^{-A_{790}})}$$

h is the heat transfer coefficient; s is the surface area of the container, T_{max} is the maximum temperature reached by the solution, T_{surr} stands for the ambient (room) temperature (T_{surr} = 25°C), Q_{Dis} represents heat dissipated from the laser mediated by the solvent and container. I is the laser power (I = 0.5 W/cm^2) and A is the absorbance at 790 nm (A_{790} = 0.5). The formulas for calculating Q_{Dis} (Q_{Dis} = 2.1) and hS are as follows (hS = 0.34):

$$Q_{Dis} = \frac{m_D C_D \Delta T_{Max(water)}}{\tau_{water}}$$

$$hS = \frac{mC_D}{\tau_S}$$

m_D is the mass of the solution containing the photoactive material, C_D is the specific heat capacity of water (C_{water} = 4.2 J/(g °C)), τ_s is the associated time constant.

$$\tau_S = \frac{-\ln(\theta)}{t}, \quad \theta = \frac{T - T_{surr}}{T_{max} - T_{surr}}$$

7. In vitro PA imaging

10 μM solution of IR780-PDA@PLX was prepared and used as the test sample. To evaluate its response to varying levels of GSH, 1 mL of IR780-PDA@PLX was mixed with GSH at different concentration ratios, followed by thorough shaking and

incubation at room temperature for 1 h. After incubation, a small aliquot was withdrawn and subjected to PA measurement using a PA imaging system to record signals at excitation wavelengths of 640 nm and 790 nm.

In a separate time-dependent study, 2 mL of the 10 μM IR780-PDA@PLX solution was reacted with GSH at a concentration ratio of 1:100. The PA signals at 640 nm and 790 nm were measured at various time points (0, 3, 6, 9, and 12 h) using the same PA imaging system to monitor the kinetics of the GSH-triggered response.

8. Cell culture

4T1 cells were cultured in DMEM containing 10% (v/v) fetal bovine serum (FBS) and 1% abtibiotics (penicillinstreptomycin, 10000 U mL^{-1}) in a humidified incubation chamber at 37°C and 5% CO_2. Same culturing process was conducted on 4T1 cells (5×10^6 cells per well), which were subcutaneously injected into orthotopic breast of the mice to establish the tumor model.

9. In vitro cytotoxicity testing

The cytocompatibility and photothermal therapeutic efficacy of IR780-PDA@PLX were evaluated using a standard Cell Counting Kit-8 (CCK-8) assay in tumor cells cultured in DMEM supplemented with varying concentrations of IR780-PDA@PLX. Cells were passaged and seeded into two 96-well plates and incubated at 37 °C with 5% CO_2 for 24 h to allow for cell attachment and proliferation. Subsequently, both plates were treated with IR780-PDA@PLX at different concentrations for 2 h. After incubation, the culture medium was removed, and the cells were gently washed with PBS. Fresh medium was then added for continued culture. One of the plates was incubated for an additional 24 h before CCK-8 reagent was added. After incubation with CCK-8 for 2 h, the absorbance at 450 nm was measured using a microplate reader to determine cell viability, thereby assessing the cytocompatibility of IR780-PDA@PLX. For the second plate, following the initial 2-hour incubation with IR780-PDA@PLX and subsequent PBS washing, the cells were irradiated with an 808 nm laser at a power density of 0.5 W/cm^2 for 10 min. After irradiation, the culture medium was replaced with fresh DMEM for further incubation. At the end of the post-

irradiation culture period, CCK-8 reagent was added, and absorbance at 450 nm was recorded to evaluate the photothermal-induced cytotoxicity of IR780-PDA@PLX.

10. Apoptosis assay

Cells were passaged and seeded into four confocal culture dishes, followed by incubation at 37 °C for 24 h. Subsequently, the culture medium was removed, and the cells were gently washed with phosphate-buffered saline (PBS). Two dishes were replenished with fresh medium containing PBS (control group), while the remaining two dishes received medium supplemented with IR780-PDA@PLX at four times the half-maximal inhibitory concentration (4×IC50). After 2 h of incubation, one dish from each group was washed with PBS and stained with a Calcein AM/propidium iodide (PI) dual-staining solution. The dishes were then incubated in the dark at 37 °C for 30 min. The other two dishes underwent 808 nm laser irradiation at a power density of 0.5 W/cm^2 for 10 min. Then the cells were stained with Calcein AM/PI probe for 30 min. Cells were washed with PBS again, and then 1 mL of fresh DMEM was added to the cells. Finally, the fluorescence was analyzed by CLSM.

11. Animal model

Healthy female Balb/c mice (6-8 weeks) were selected for the establishment of the tumour model. 4T1 cells were used to establish an in-situ breast cancer mouse model. After the tumors grew for about 10 days to reach a volume of about 60-80 mm^3, they were used for photoacoustic and photothermal imaging and photosensitization experiments.

12. In vivo photothermal evaluation

Following hair removal from the mouse legs, the animals were continuously anesthetized using inhaled anesthetic gas. The tumor sites on the legs were then irradiated with an 808 nm laser at a power density of 0.5 W cm^{-2} for 10 min. An infrared thermal imaging camera was used throughout the procedure to monitor and record temperature changes at the tumor sites.

13. In vivo PA imaging

IR780-PDA@PLX (200 μL) was administered to mice via tail vein injection. Following injection, the mice were anesthetized continuously and fixed in position for imaging. In vivo PA imaging was performed at predetermined time points (0, 3, 6, 9, and 12 h) using a photoacoustic imaging system. PA signals were recorded to evaluate the temporal distribution of the nanomaterial in the tumor site and to determine the optimal time point for tumor accumulation and NIR absorption activation.

14. In vivo treatment

4T1 tumor-bearing mice were randomly divided into 2 groups (5 mice/group), and 2 comparative treatment groups were set up: (1) control group (PBS); (2) IR780-PDA@PLX + 808 nm laser (0.5 W cm^{-2}). Twelve hours after the injection, the tumor site was irradiated with 808 nm laser for 10 min (0.5 W cm^{-2}), and the warming process of the tumor site was recorded by infrared thermography. At the end of the treatment, all 4T1 tumor-bearing mice were dissected and analyzed by removing the intact tumor tissue, the major organs (heart, liver, spleen, lungs, kidneys) and the skin tissue at the light-exposed site of the tumor. The excised tumors were sequentially placed in groups, photographed and then weighed separately. Tumor tissues and organs were fixed with 4%PFA. Subsequently, paraffin-embedded tissue sections were stained with hematoxylin and eosin (H&E) to analyses the cellular status of the tumor tissues and major organs. At the same time, the tumor skin tissue in the laser irradiated area was processed by hematoxylin and eosin (H&E).

15. Ethics approval

All animal experiments were performed with the permission of the Animal Ethics Committee of Jiangsu Keygen Biotech Co., Ltd. (China), according to the guidelines approved by the Jiangsu Administration of Experimental Animals (Approval Number: IACUC-002-24).

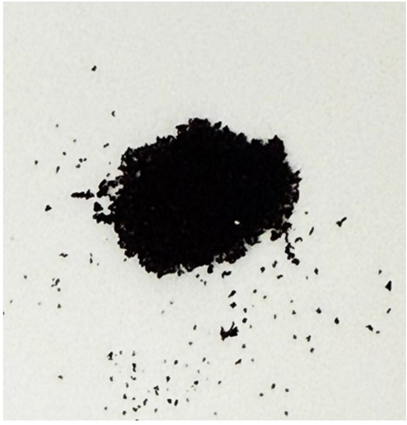

Fig. S1 The physical pictures of IR780-PDA@PLX.

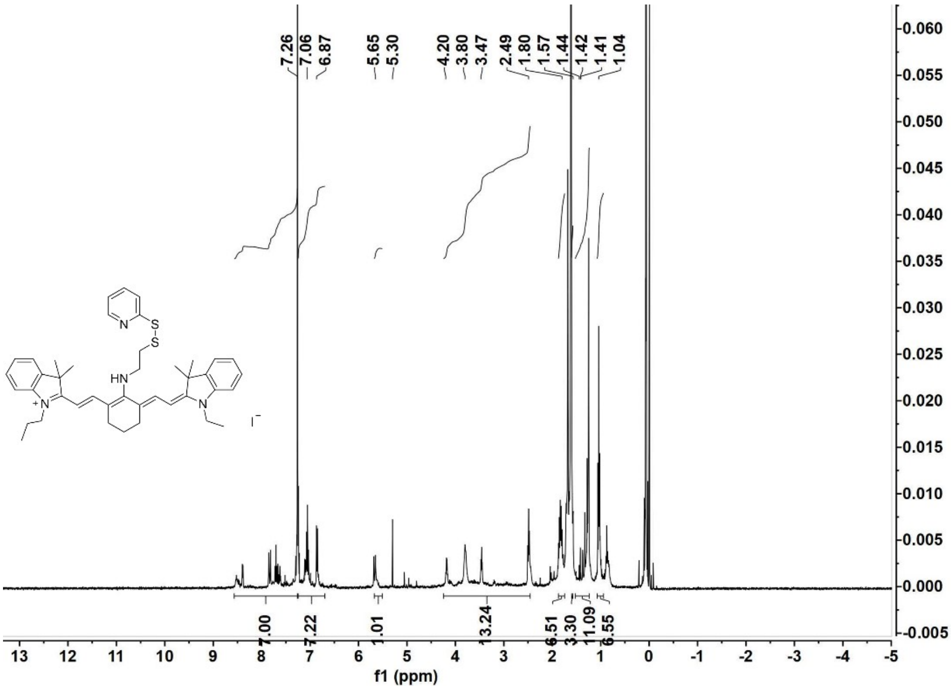

Fig. S2 ^{1}H-NMR spectrum of IR780-PDA.

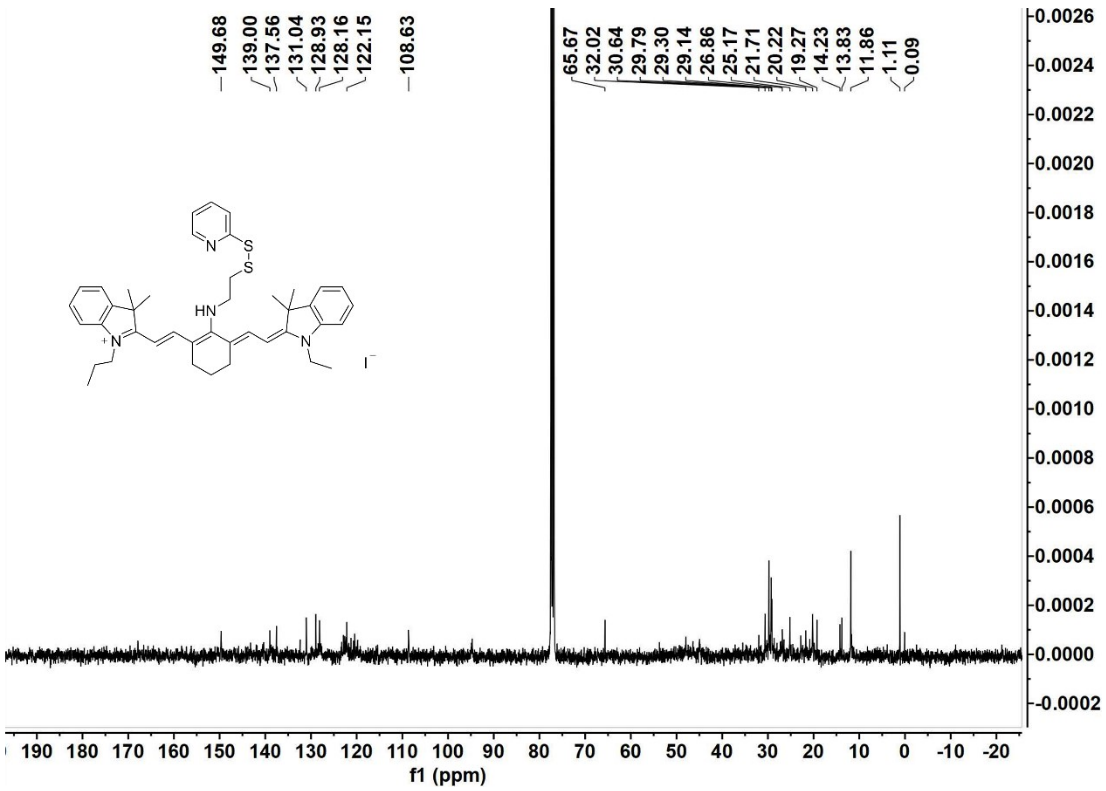

Fig.S3 ^{13}C-NMR spectrum of IR780-PDA.

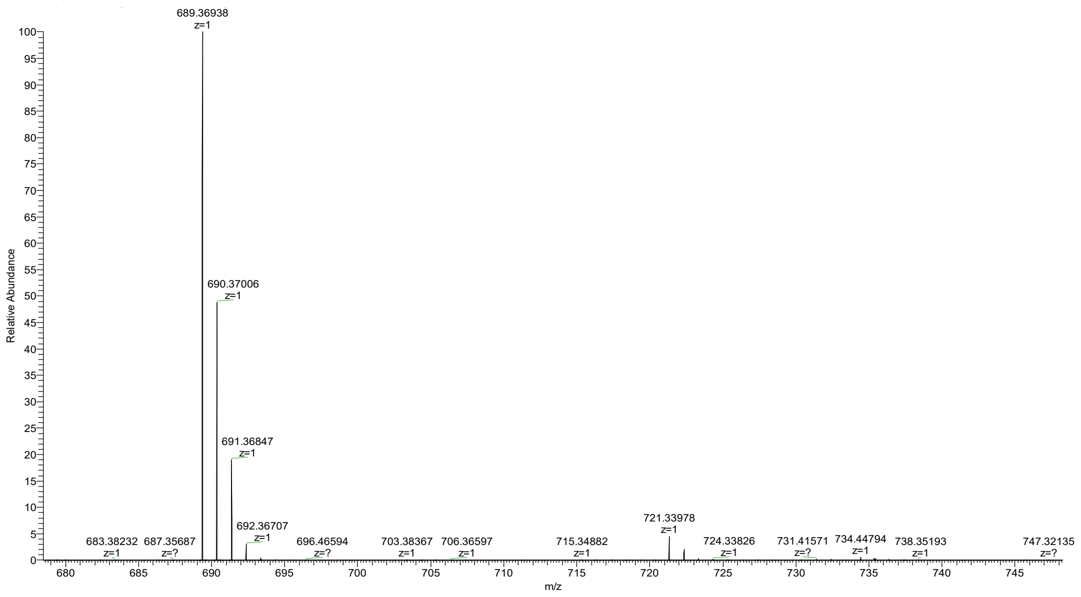

Fig. S4 HR-MS of IR780-PDA.

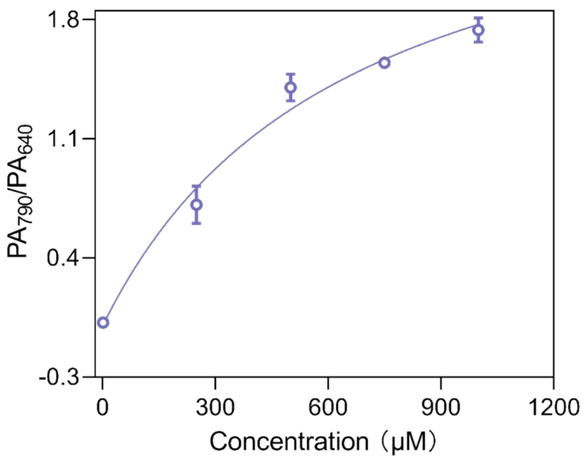

Fig. S5 Relationship between ratio absorbance intensity (Ab_{790}/Ab_{640}) and GSH concentration.

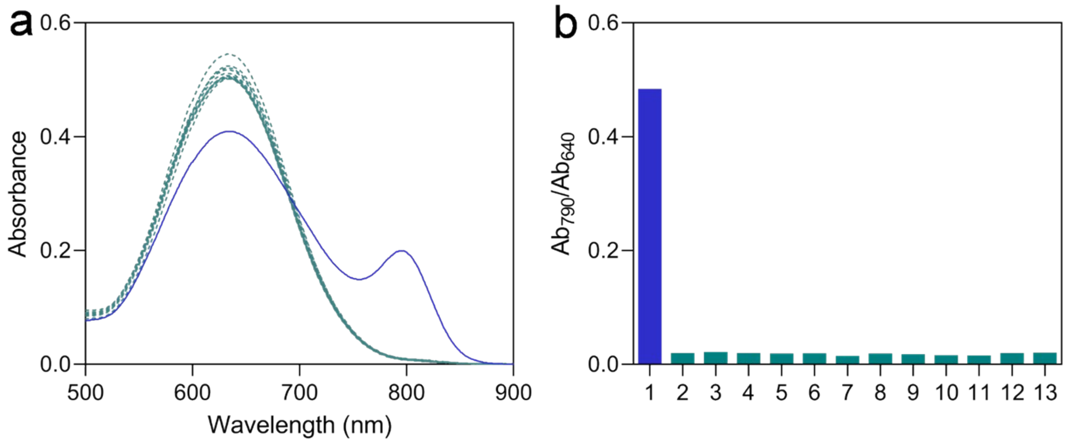

Fig. S6 (a) Selectivity spectra of IR780-PDA@PLX with different amino acids and glutathione. (b) The ratiometric absorption intensities (Ab_{790}/Ab_{640}) of IR780-PDA@PLX in different analytes (1-13 represent GSH, tyrosine, phenylalanine, histidine, serine, alanine, proline, ornithine, lysine, valine, arginine, isoleucine, and aspartic acid).

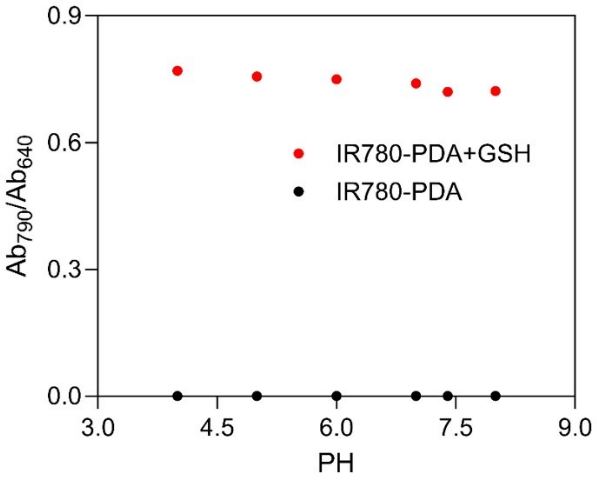

Fig. S7 Response of IR780-PDA@PLX to the reaction with GSH under different pH conditions.

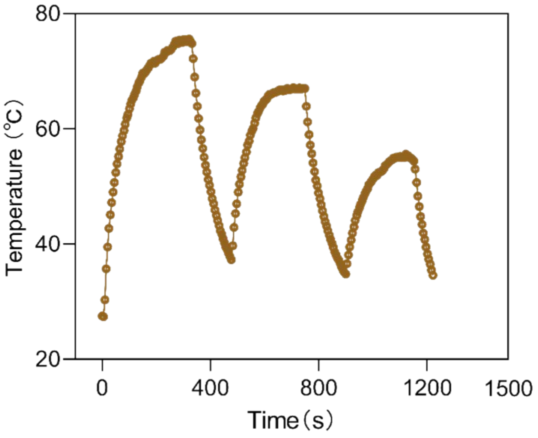

Fig. S8 The heating and cooling cyclic curves of GSH activated IR780-PDA@PLX under 808 nm laser irradiation.

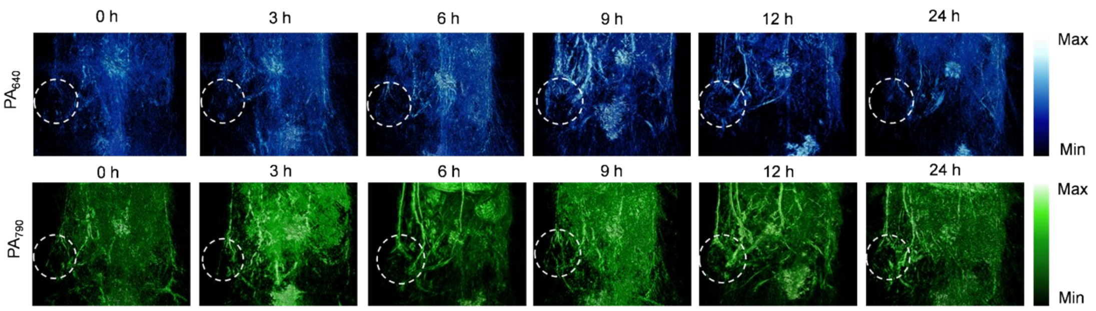

Fig. S9 PA images of 4T1 tumours in nude mice before and after systemic injection of IR780-PDA@PLX (0, 3, 6, 9, 12, and 24 h).

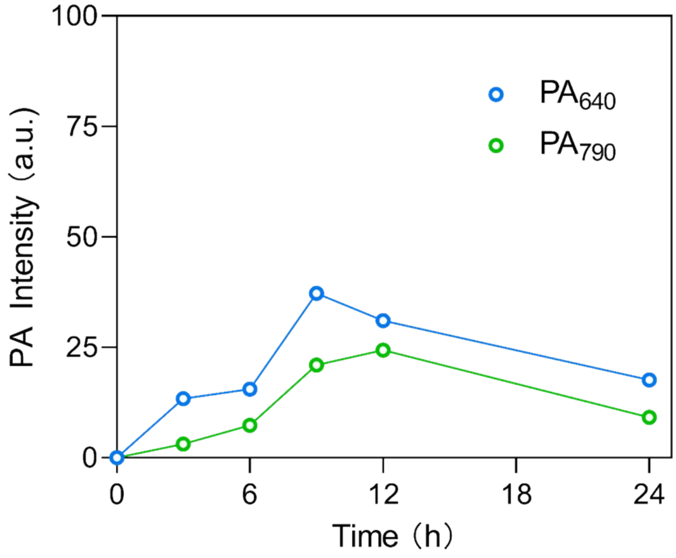

Fig. S10 Ratiometric PA intensities (PA_{790}/PA_{640}) as a function of postinjection time.

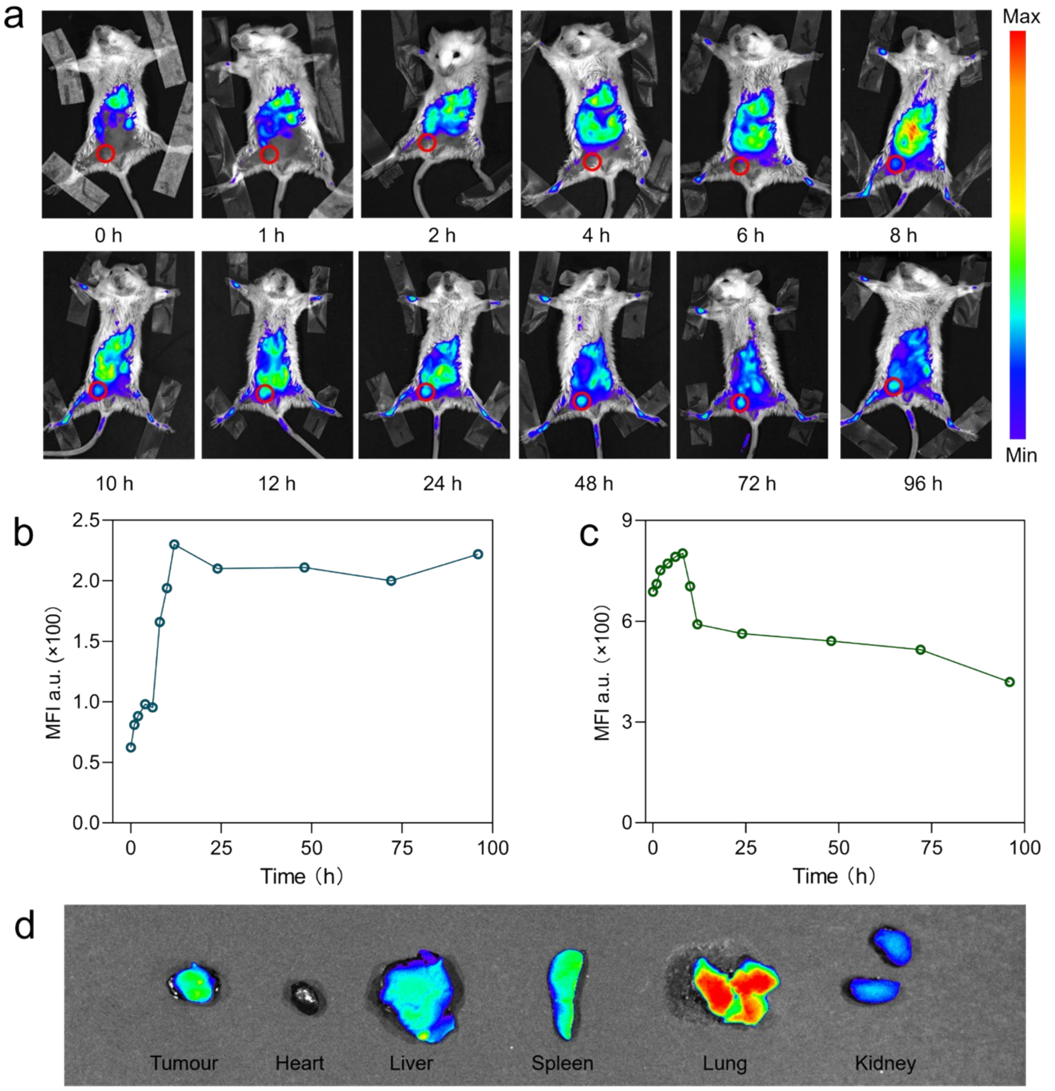

Fig. S11 (a) In vivo fluorescence images within 96 hours after intravenous injection of IR780-PDA@PLX. (b) Time-dependent fluorescence intensity of IR780-PDA@PLX at the tumor site. (c) Time-dependent fluorescence intensity of IR780-PDA@PLX in the liver. (d) Ex vivo fluorescence images of tumors and major organs harvested from mice.

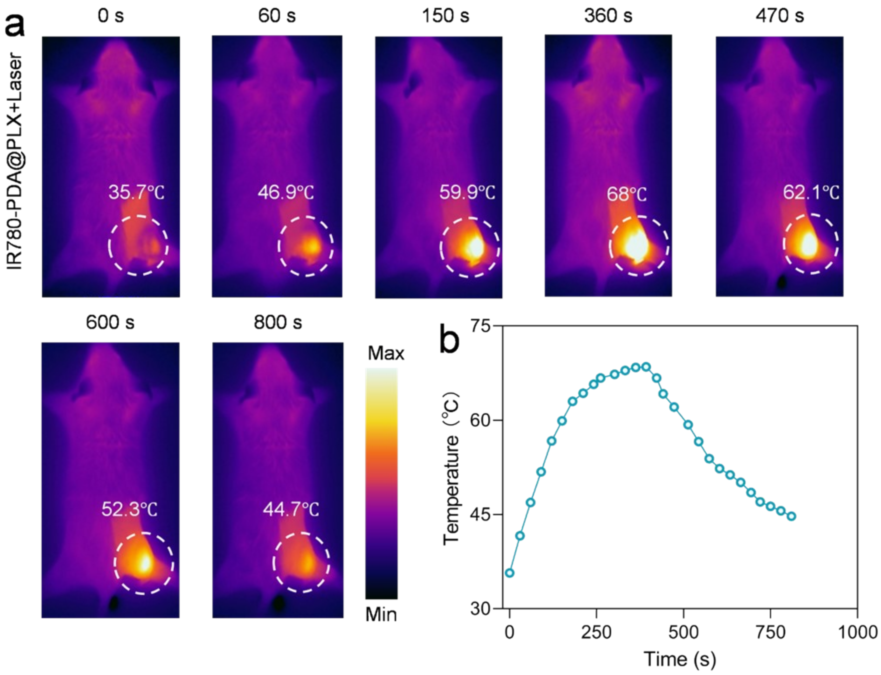

Fig. S12 (a) Thermal imaging of tumor site of mice following IR780-PDA@PLX injection under laser irradiation (0.5 W cm^{-2}) within 800 s. (b) Time-dependent temperature rise curve at the tumor site in mice following IR780-PDA@PLX injection and 808 nm laser irradiation (0.5 W cm^{-2}).

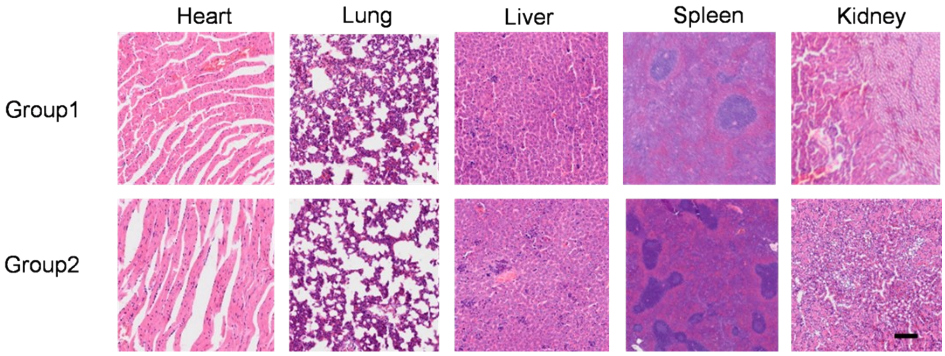

Fig. S13 H&E stained images of mouse tumor tissues (heart, liver, spleen, lung and kidney) after PTT. Scale bar: 200 μm.

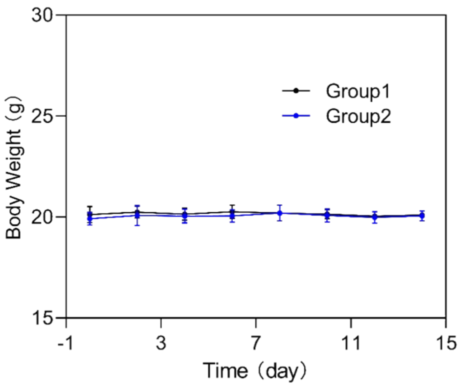

Fig. S14 Body weight change curves of 4T1 tumor-bearing mice after various treatments.

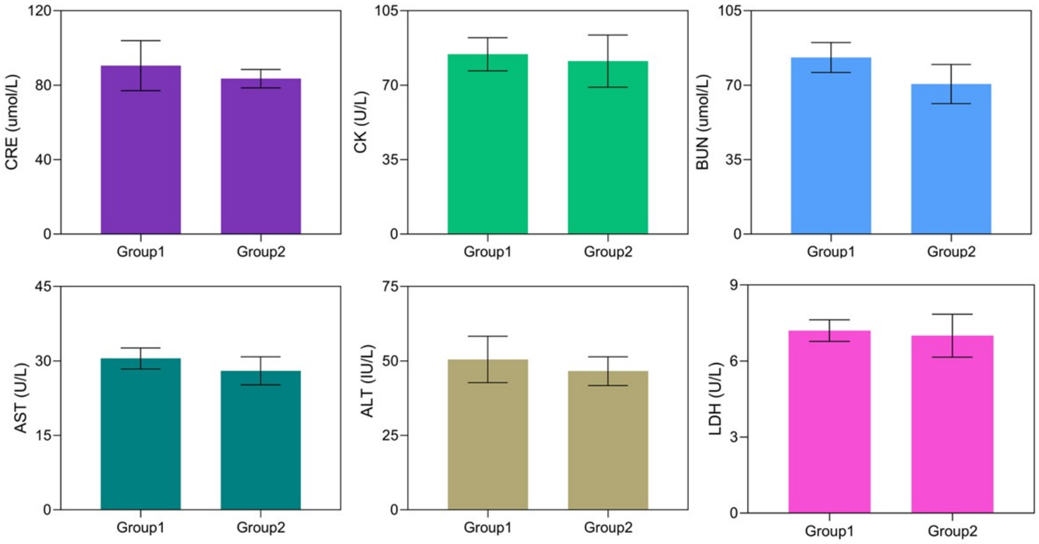

Fig. S15 Chemical analysis of blood biochemical indices in two groups of mice after different treatments.

Table S1 Comparative analysis of TME-responsive photothermal nanomaterials

Kind	Activation	PCE	Degradation	Therapeutic Efficacy
IR780-PDA@PLX	GSH	43.65%	Photodegradation; cleared via kidneys and livers	Tumor complete regression
LET-5[1]	pH	44.6%	Biodegradable RBC vesicles, cleared by reticuloendothelial	Full tumor regression, no recurrence

Kind	Activation	PCE	Degradation	Therapeutic Efficacy
			system	
b-P25@MnO_2[2]	GSH	30.67%	Cleared via kidneys and livers	Tumor eradication, prolonged survival
Hypoxia-vesicles [3]	Hypoxia	19.9%	Disassemble under hypoxia	Tumor growth inhibition, prolonged survival
IR780@SAC4A[4]	Hypoxia	/	Cleared via kidneys and livers	Significant tumor volume reduction
PPDI-NPs[5]	pH	45.3%	Cleared via kidneys and livers	Full tumor regression, prolonged survival
AZGL[6]	pH	27.5%	Degraded in acidic TME	Tumor ablation and amelioration of hypoxia
IR&DOX@NC[7]	GSH	69.3%	Cleared via kidneys and livers	98.7% tumor suppression
BPN/MnO_2[8]	H_2O_2/GSH	32.7%	Cleared via kidneys and livers	Tumor growth inhibition

Reference:

1 X. Gao, S. Jiang, C. Li, Y. Chen, Y. Zhang, P. Huang and J. Lin, *Biomaterials,* 2021, **267**, 120454.

2 Y. Gao, Z. Yin, Q. Ji, J. Jiang, Z. Tao, X. Zhao, S. Sun, A. Wu and L. Zeng, *J. Mater. Chem. B*, 2021, **9**, 314-321.

3 Q. Yu, L. Tu, T. Zhu, H. Zhu, S. Liu, Y. Sun and Q. Zhao, *ACS Appl. Mater. Interfaces*, 2022, **14**, 50637-50648.

4 H. Keum, E. Cevik, J. Kim, Y. M. Demirlenk, D. Atar, G. Saini, R. A. Sheth, A. R. Deipolyi and R. Oklu, *Adv. Mater.*, 2024, **36**, 2310856.

5 J. Li, C. Liu, Y. Hu, C. Ji, S. Li and M. Yin, *Theranostics*, 2020, **10**, 166-178.

6 R.-T. Li, Y.-D. Zhu, W.-Y. Li, Y.-K. Hou, Y.-M. Zou, Y.-H. Zhao, Q. Zou, W.-H. Zhang and J. X. Chen, *J Nanobiotechnol*, 2022, **20**, 212.

7 D. Cheng, Y. Ji, B. Wang, Y. Wang, Y. Tang, Y. Fu, Y. Xu, X. Qian and W. Zhu, *Acta Biomaterialia*, 2021, **128**, 435-446.

8 Q. Wu, G. Chen, K. Gong, J. Wang, X. Ge, X. Liu, S. Guo and F. Wang, *Matter*, 2019, **1**, 496-512.
